# Supplementary material for: Complex‐centric proteome profiling by SEC‐SWATH‐MS
Source: Mol Syst Biol. 2019 Jan 14;15(1):e8438. doi: 10.15252/msb.20188438 (PMC6346213; doi:10.15252/msb.20188438)
Supplement: Supplementary file 8 — Dataset EV7 [file MSB-15-e8438-s008.zip › feature_plots_string/Q9NVS2.pdf]

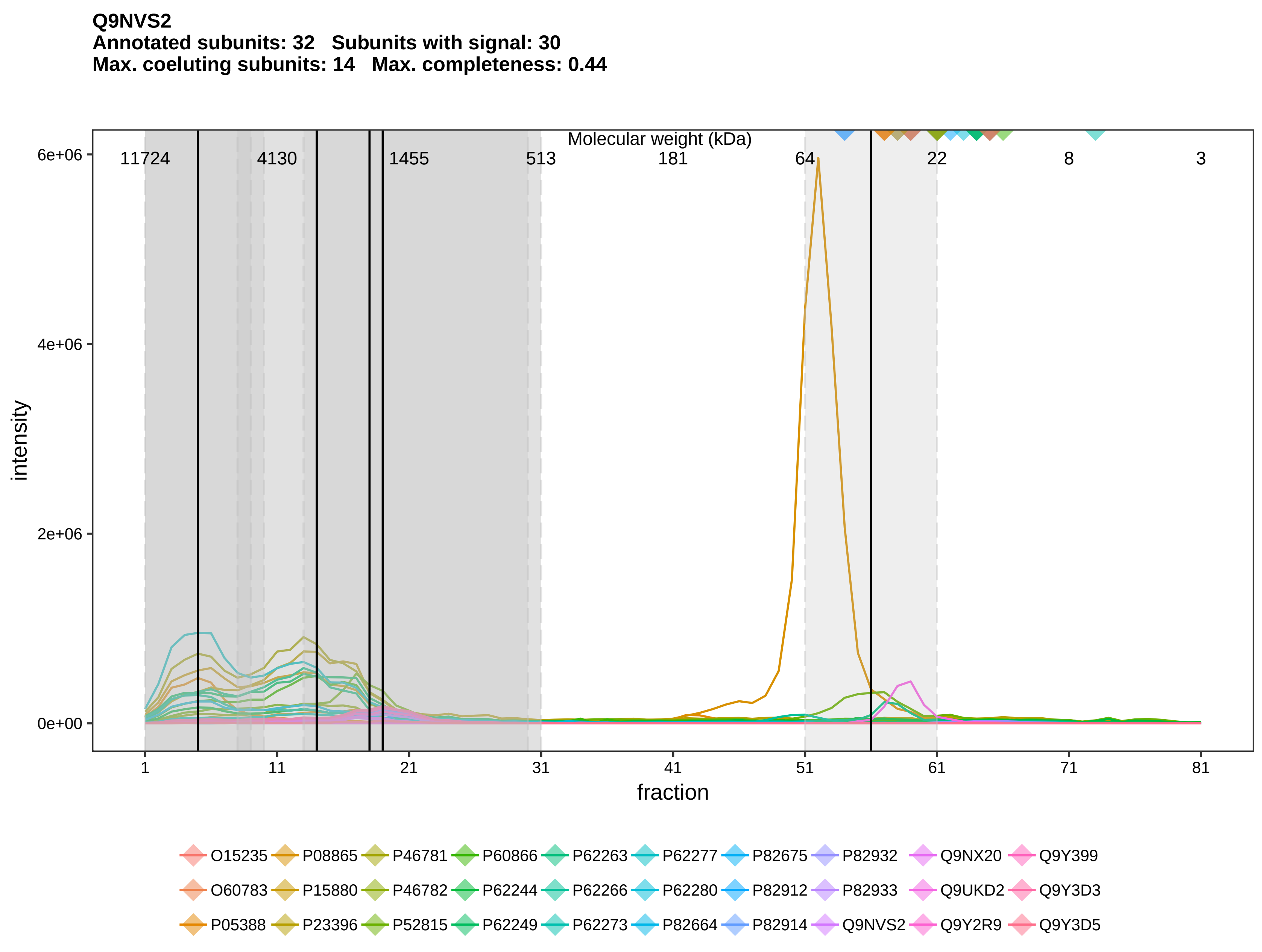

Q9NVS2
Annotated subunits: 32 Subunits with signal: 30
Max. coeluting subunits: 14 Max. completeness: 0.44
Molecular weight (kDa)
11724
4130
1455
513
181
64
22
8
3
6e+06
4e+06
2e+06
0e+00
intensity
1
11
21
31
41
51
61
71
81
fraction
O15235
P08865
P46781
P60866
P62263
P62277
P82675
P82932
Q9NX20
Q9Y399
O60783
P15880
P46782
P62244
P62266
P62280
P82912
P82933
Q9UKD2
Q9Y3D3
P05388
P23396
P52815
P62249
P62273
P82664
P82914
Q9NVS2
Q9Y2R9
Q9Y3D5
